# The MUK eight protocol: a randomised phase II trial of cyclophosphamide and dexamethasone in combination with ixazomib, in relapsed or refractory multiple myeloma (RRMM) patients who have relapsed after treatment with thalidomide, lenalidomide and a proteasome inhibitor

**DOI:** 10.1186/s13063-020-04739-8

**Published:** 2020-10-02

**Authors:** Samantha Hinsley, Katrina Walker, Debbie Sherratt, Lucy Bailey, Sadie Reed, Louise Flanagan, Sophie McKee, Fiona Brudenell Straw, Bryony Dawkins, David Meads, Holger W. Auner, Martin F. Kaiser, Mark Cook, Sarah Brown, Gordon Cook, on behalf of the Myeloma UK Clinical Trials Network

**Affiliations:** 1 Clinical Trials Research Unit, Leeds Institute of Clinical Trials Research, University of Leeds, Leeds, UK; 2 Academic Unit of Health Economics, Leeds Institute of Health Sciences, University of Leeds, Leeds, UK; 3 Centre for Haematology, Imperial College London, London, UK; 4 Division of Molecular Pathology, The Institute of Cancer Research, London, UK; 5 Centre for Haematology, University Hospitals Birmingham NHS Foundation Trust, Birmingham, UK; 6 Leeds Institute of Cancer and Pathology, University of Leeds, Leeds, UK

## Abstract

**Background:**

Multiple myeloma is a plasma cell tumour with approximately 5500 new cases in the UK each year. Ixazomib is a next generation inhibitor of the 20S proteasome and is thought to be an effective treatment for those who have relapsed from bortezomib. The combination of cyclophosphamide and dexamethasone (CD) is a recognised treatment option for patients with relapsed refractory multiple myeloma (RRMM) who have relapsed after treatment with bortezomib and lenalidomide, whilst also often being combined with newer proteasome inhibitors. The most apparent combination for ixazomib is therefore with CD.

**Methods:**

MUK eight is a randomised, controlled, open, parallel group, multi-centre phase II trial that will recruit patients with RRMM who have relapsed after treatment with thalidomide, lenalidomide, and a proteasome inhibitor. The primary objective of the trial is to evaluate whether ixazomib in combination with cyclophosphamide and dexamethasone (ICD) has improved clinical activity compared to CD in terms of progression-free survival (PFS). Secondary objectives include comparing toxicity profiles and the activity and cost-effectiveness of both treatments. Since opening, the trial has been amended to allow all participants who experience disease progression (as per the IMWG criteria) on the CD arm to subsequently switch to receive ICD treatment, once progression has been confirmed with two clinical members of the Trial Management Group (TMG). This ‘switch’ phase of the study is exploratory and will assess second progression-free survival measured from randomisation to second disease progression (PFS2) and progression-free survival from the point of switching to second disease progression (PFS Switch) in participants who switch from CD to ICD treatment.

**Discussion:**

Development of ixazomib offers the opportunity to further investigate the value of proteasome inhibition through oral administration in the treatment of RRMM. Previous studies investigating the safety and efficacy of ICD in patients with RRMM demonstrate a toxicity profile consistent with ixazomib in combination with lenalidomide and dexamethasone, whilst the combination showed possible activity in RRMM patients. Further investigation of the anti-tumour effect of this drug in RRMM patients is therefore warranted, especially since no trials comparing CD with ICD have been completed at present.

**Trial registration:**

ISRCTN number: ISRCTN58227268. Registered on 26 August 2015.

## Background

Multiple myeloma (MM) is a malignancy of plasma cells, characterised by proliferation of these cells in the bone marrow and, usually, abnormal secretion of monoclonal immunoglobulins. In 2015, there were 5540 new cases of MM in the UK, an age-standardised incidence of 9.3 per 100,000 population in patients with a new cancer diagnosis, and in 2014, there were 2928 deaths from the disease [1]. Survival rates for MM have improved significantly in recent decades, with 77% of patients in England and Wales currently surviving for at least 1 year following diagnosis, compared to 37% 40 years ago. Ten-year survival rates have increased from 6 to 33% in the same time period [1]. Despite these improvements, which can be attributed to advances in both diagnostics and treatment, MM is incurable and the majority of patients will at some point require further therapy for relapsed disease.

Treatment options for relapsed patients include both conventional cytotoxic agents, newer immunomodulatory agents and proteasome inhibitors, and autologous stem cell transplantation (ASCT). As with treatment for primary disease, a combination of treatments is commonly employed. However, many patients are either refractory to treatment using existing approved regimens, or will relapse after an initial response. Investigation of further treatment options for these patients is therefore required.

Ixazomib is a next generation, small molecule inhibitor of the 20S proteasome that is under development for the treatment of non-haematological malignancies, lymphoma, MM, and other plasma cell dyscrasias. Inhibition of the 20S proteasome has previously been validated as a therapeutic target for the treatment of malignancies using bortezomib [2]; however, some patients do not respond to bortezomib or develop resistance to this drug. Ixazomib is structurally and pharmacologically distinct from bortezomib and is orally bioavailable. Furthermore, it has already been shown to be well-tolerated, with a favourable toxicity profile and promising efficacy [3–6].

**Fig. 1:**
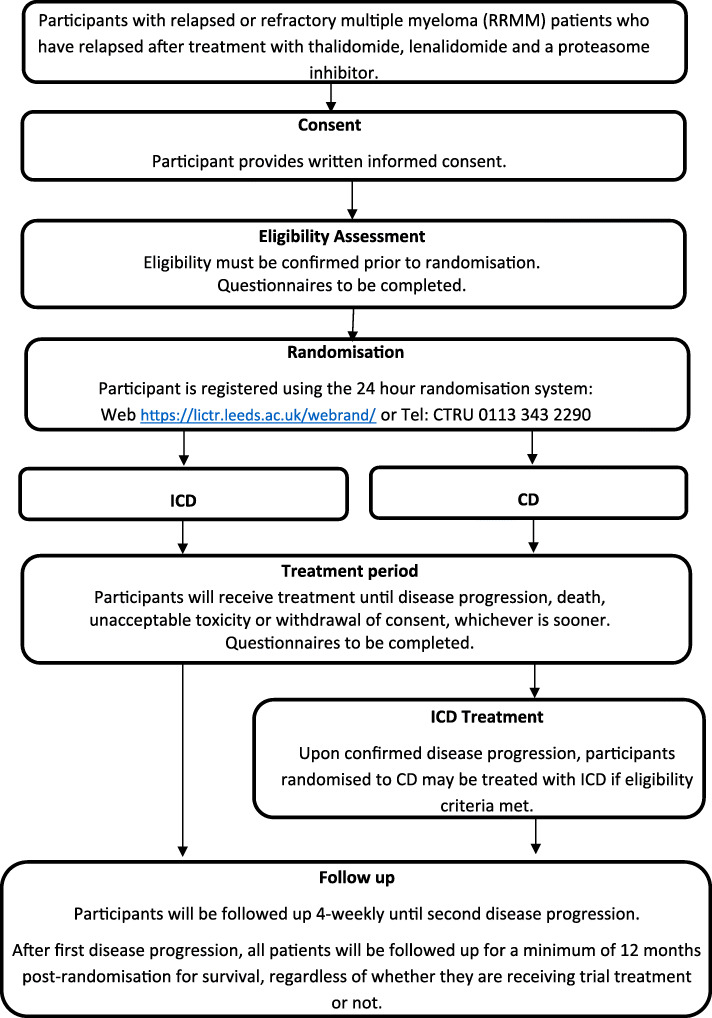
Trial flow diagram

The Myeloma UK (MUK) study, MUK eight, has been developed to further investigate the potential of ixazomib for treatment of relapsed/refractory multiple myeloma (RRMM) (Fig. 1). For patients with RRMM who have relapsed after treatment with thalidomide, lenalidomide and a proteasome inhibitor, the combination of cyclophosphamide and dexamethasone (CD) is a recognised treatment option alternative to a novel therapy. CD is also already regularly used, to good effect, in combination with newer agents including bortezomib. Therefore in the RRMM setting, the most apparent combination for ixazomib is with CD.

The study has been developed through the MUK Early Phase Clinical Trials Network (CTN), an innovative collaboration which brings together clinical specialists and researchers, the pharmaceutical industry and NHS regulatory bodies to conduct a prioritised and strategic portfolio of myeloma clinical trials [7].

Current protocol: V3.0, 23/05/2017.

## Methods

### Study aim

The MUK eight study will evaluate the clinical effectiveness of ixazomib in combination with cyclophosphamide and dexamethasone (ICD), as compared to the combination of cyclophosphamide and dexamethasone alone (CD), in terms of progression-free survival (PFS): the time from randomisation to first disease progression. MUK eight will recruit patients with RRMM who have relapsed after treatment with thalidomide, lenalidomide and a proteasome inhibitor.

### Study design

MUK eight is designed as a randomised, controlled, open, parallel group, multi-centre phase II trial. A maximum of 250 participants will be recruited and randomised on a 1:1 basis to receive either ICD or CD.

Since opening, in order to increase appeal of the control arm, the trial has been amended to allow all participants who experience disease progression (as per the IMWG criteria) on the CD arm to subsequently switch to receive ICD treatment, once progression has been confirmed by two clinical members of the Trial Management Group (TMG).

**Fig. 2:**
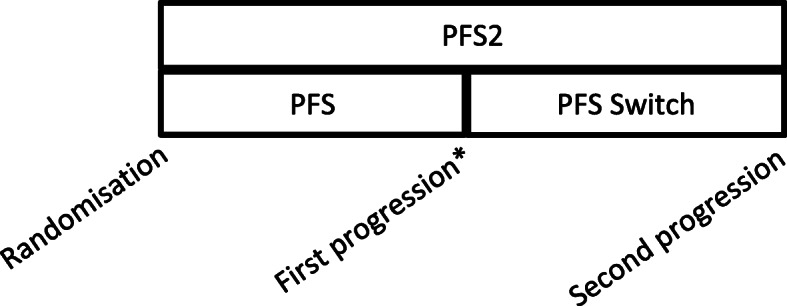
Explanation of PFS endpoints. *After patients randomised to the CD arm reach disease progression, they are given the option to switch to ICD treatment until they disease progress a second time. Patients randomised to the ICD arm that reach disease progression will be followed-up but treated off-trial as per standard practice

This phase will assess second progression-free survival measured from randomisation to second disease progression (PFS2) and progression-free survival from the point of switching to second disease progression (PFS Switch), as detailed in Fig. 2, in participants who switch from CD to ICD treatment. Participants who progress on the ICD arm will be followed up but treated off-trial as per standard practice.

### Primary objective

To evaluate whether ICD has improved clinical activity compared to CD in terms of progression-free survival (PFS), in patients with RRMM who have relapsed after treatment with thalidomide, lenalidomide and a proteasome inhibitor.

### Secondary objectives

- To further evaluate the clinical activity of ICD with regard to additional secondary endpoints, detailed in Table 3
- To determine the safety and toxicity profile of ICD compared to CD
- To estimate the cost-effectiveness of ICD compared to CD
- To determine quality of life with ICD compared to CD
- To assess the impact of baseline Charlson index score on PFS, overall survival and deliverability of planned treatment

### Exploratory objectives

- To identify possible biomarkers of response to ICD
- To estimate second progression-free survival (PFS2) measured from randomisation to second disease progression for those that switch to ICD treatment following CD treatment
- To estimate progression-free survival from the point of switching to second disease progression (PFS Switch) for those that switch to ICD treatment following CD treatment
- To evaluate the clinical activity of ICD with regard to additional exploratory endpoints, detailed in Table 3
- To determine the safety and toxicity profile of ICD after progressing on CD treatment

### Sample size

Using data from previous studies, median PFS in the CD arm is expected to be in the region of 4 to 6 months whilst median overall survival (OS) in the CD arm is expected to be around 8 months [8–13]. It is anticipated that the addition of ixazomib to CD (ICD), in RRMM patients who have relapsed after treatment with thalidomide, lenalidomide and a proteasome inhibitor, may improve clinical efficacy by at least 50%, in terms of median PFS. One formal interim efficacy analysis is planned for when 70% of the PFS events (139 events) have been observed, to assess for superiority of ICD.

**Fig. 3:**
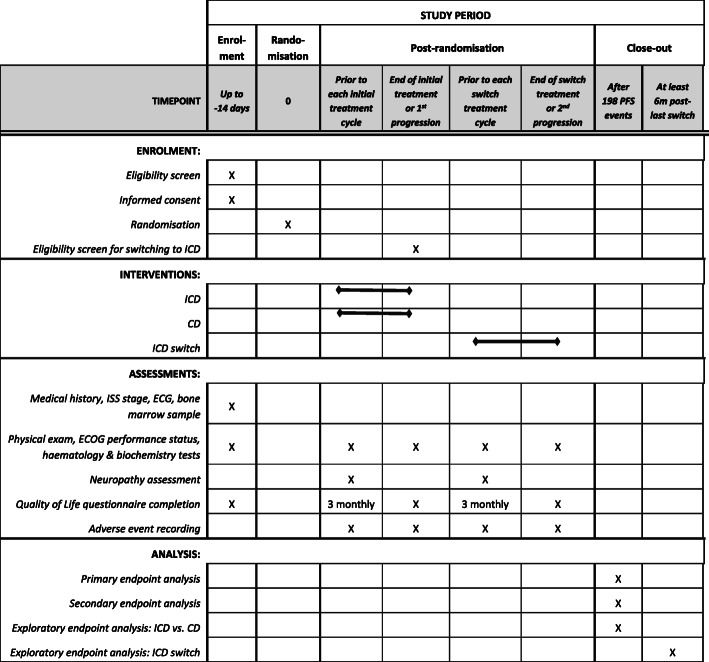
SPIRIT figure: schedule of enrolment, interventions, assessments and analysis

For the final analysis, assuming median PFS of 6 months in the CD arm, a total of 198 PFS events are required to detect an improvement in median PFS to 9 months with the addition of ixazomib to CD, corresponding to a hazard ratio of 0.67 (80% power, 4.55% 2-sided significance level in order to maintain 5% significance overall when taking into account the interim analysis). A total of approximately 230 patients (115 patients per arm) will be needed for patient enrolment to generate a total of 198 PFS events (Fig. 3). This assumes recruitment over a 24-month period, with an additional minimum 12 months’ follow-up. Allowing for dropout of 8%, a total of 250 participants are required. With 250 patients generating more than a total of 198 PFS events, this will also provide sufficient power (> 80%) to detect an improvement of 50% in median PFS where the control arm median PFS is anywhere in the region of 4 to 6 months.

For the interim analysis, the O’Brien and Fleming [14] approach will be used in order to maintain an overall 5% 2-sided significance level when performing a single interim analysis before final analysis. Superiority at the interim analysis, in terms of PFS, will therefore be tested at a 0.0074 1-sided significance level, whilst the final analysis will be tested at a 0.0455 2-sided significance level.

At the interim analysis, assuming median PFS of 6 months in the CD arm, there will be approximately 47% power to detect a hazard ratio of 0.67. A low power has been used as an early release of data will only take place at this point if an overwhelming treatment effect is seen.

A total of 186 deaths, which are expected from the sample size of 250, provide over 80% power to detect an improvement in median overall survival (OS) from 8 months with CD to 11.5 months with ICD, corresponding to a hazard ratio of 0.7, at the 2-sided 10% significance level.

This ‘switch’ phase of the study is exploratory so no formal sample size calculation has been performed.

### Recruitment process

When calculating the sample size, the trial was expected to take 24 months to complete recruitment, at a rate of 15 participants per month, and the first trial participant was randomised on 21 January 2016. However, due to slower recruitment than anticipated, it is likely the trial will take longer than this to complete recruitment. Participants will be recruited from NHS hospitals throughout the UK which are approved research sites within the MUK Early Phase CTN. A full list of study sites can be accessed via the trial registration, ISRCTN58227268.

Research sites will be required to have obtained ethical and management approval and undertake a site initiation meeting with Clinical Trials Research Unit (CTRU) prior to opening to recruitment. Potential participants will be approached by authorised members of staff, usually during standard clinic visits for management of their disease, who will verbally describe and discuss the trial and then provide the patient information sheet.

Participants will be given as long as they need to consider whether they wish to take part and will be able to discuss the study with their family, friends and other healthcare professionals if they wish. Consenting participants will be invited to provide informed, written consent and will be formally assessed for eligibility, according to the inclusion and exclusion criteria detailed in Table 1.

**Table 1:** MUK eight study inclusion and exclusion criteria

**Inclusion criteria**	
●Able to give informed consent and willing to follow study protocol assessments ●Aged 18 years or over ●Participants with confirmed MM based on IMWG criteria, 2009 ●Measurable disease with at least one of the following: ○Paraprotein > 5 g/L or 0.5 g/l for IgD subtype ○Serum free light chains > 100 mg/L with abnormal ratio for light chain only myeloma ○Bence Jones protein > 200 mg/L ●Participants with relapsed or relapsed refractory myeloma and now require further treatment following exposure to thalidomide, lenalidomide and a proteasome inhibitor regardless of response to these. ●Participants for which CD would be a suitable treatment ●ECOG Performance Status ≤ 2 ●Required laboratory values within 14 days prior to start of treatment: ○Platelet count ≥ 50 × 10^9^/L. Platelet count of 30–50 is acceptable if bone marrow aspirate shows tumour replacement of > 50%. Platelet support is permitted within 14 days prior to randomisation although platelet transfusions to help patients meet eligibility criteria are not allowed within 72 h prior to the blood sample to confirm protocol eligibility. ○Absolute neutrophil count ≥ 1.0 × 10^9^/L. Growth factor support is **not** permitted within 14 days prior to randomisation ○Haemoglobin > 90 g/L. Blood support is permitted ○ALT and / or AST ≤ 3 × upper limit of normal ○Creatinine clearance ≥ 30 ml/min (using Cockcroft Gault formula) ○Bilirubin ≤ 1.5 × upper limit of normal ●Female participants should avoid becoming pregnant and male participants should avoid impregnating a female partner. Both non-sterilised and sterilised females and males of reproductive age should use effective methods of contraception during the entire trial treatment (including treatment breaks) and up to 90 days after the last dose of trial treatment. Females of child bearing potential will require a negative pregnancy test to be performed. ●Post allograft patients may be included if > 12 months from transplant.	
**Exclusion criteria**	
●Participants meeting any of the following exclusion criteria are not eligible to register for this trial. ●The following participants will be excluded: ○Those with non-measurable disease ○Those with a solitary bone or solitary extramedullary plasmacytoma ○Plasma cell leukaemia ●Prior malignancy other than those treated with curative surgery. ●Participants with a known or underlying uncontrolled concurrent illness that, in the investigators opinion, would make the administration of the study drug hazardous or circumstances that could limit compliance with the study, including, but not limited to the following: acute or chronic graft versus host disease, uncontrolled hypertension, congestive heart failure ≥ NYHA Class III, unstable angina pectoris, myocardial infarction within past 6 months, uncontrolled cardiac arrhythmia, renal failure, psychiatric or social conditions that may interfere with participant compliance, or any other condition (including laboratory abnormalities) that in the opinion of the Investigator places the participant at unacceptable risk for adverse outcome if he/she were to participate in the study. ●Participants who have previously received MLN9708/ixazomib in a trial. Previous experimental agents or approved anti-tumour treatment within 28 days before the date of randomisation. ●A maximum of 160 mg of dexamethasone (in 40 mg blocks) may be given between screening and the beginning of initial treatment if medically required but should be stopped before trial treatment starts. Bisphosphonates for bone disease are also permitted. ●Participants with a history of a refractory nausea, diarrhoea, vomiting, malabsorption, gastrointestinal surgery or other procedures that might, in the opinion of the Investigator, interfere with the absorption or swallowing of the study drug(s) ●Peripheral neuropathy of ≥ grade 2 (or grade 1 with pain) severity (as per NCI-CTCAEv4.0) ●Gastrointestinal disorders that may interfere with absorption of the study drug ●Active symptomatic fungal, bacterial, and/or viral infection including known active HIV or known viral (A, B or C) hepatitis ●Female participants who are lactating or have a positive pregnancy test at screening ●Known allergy to any of the study medications, their analogues, or excipients in the various formulations of any agent that would prevent the participant receiving these as directed in the protocol ●Systemic treatment, within 14 days prior to the first dose of ixazomib, with strong inhibitors of CYP1A2 (fluvoxamine, enoxacin, ciprofloxacin), strong inhibitors of CYP3A (clarithromycin, telithromycin, itraconazole, voriconazole, ketoconazole, nefazodone, posaconazole) or strong CYP3A inducers (rifampin, rifapentine, rifabutin, carbamazepine, phenytoin, phenobarbital), or use of *Ginkgo biloba* or St. John’s wort ●Major surgery within 14 days prior to the date of randomisation ●Radiotherapy within 7 days prior to randomisation for palliative pain control or therapeutic radiotherapy within 14 days prior to randomisation ●Myeloma involving the Central Nervous System.	

### Randomisation

Written informed consent for entry into the trial must be obtained, with eligibility being confirmed prior to randomisation. Randomisation will be administered using an automated 24-h web-based system managed by the CTRU. A computer-generated minimisation programme that incorporates a random element will be used to balance treatment groups for age (< 60 vs. 60–69 vs. ≥ 70), number of prior lines of therapy (≤ 3 vs. > 3) and β2M (< 3.5 mg/L vs. 3.5 mg/L ≤ β2M < 5.5 mg/L vs. ≥ 5.5 mg/L) at randomisation.

Study centre has not been included as a minimisation factor as the primary endpoint is standardly assessed across all centres, as seen from previous trials run through CTRU; therefore, clustering within sites will not likely be an issue. Additionally, the impact of treatment groups not being balanced within a centre is thought to be less problematic than treatment group unbalance across the minimisation factors selected.

### Intervention

Patients randomised to ICD will receive the following regimen: ixazomib (oral) 4 mg on days 1, 8 and 15; cyclophosphamide (oral) 500 mg on days 1, 8 and 15; and dexamethasone (oral) 40 mg on days 1–4 and 12–15.

Patients randomised to CD will receive the following regimen: cyclophosphamide (oral) 500 mg on days 1, 8 and 15 and dexamethasone (oral) 40 mg on days 1–4 and 12–15.

For older/less fit patients in both cohorts (as determined by the Principal Investigator), dexamethasone may be given orally at 20 mg on days 1–4 and 12–15.

In both cohorts, the cycle is repeated every 28 days with response being assessed at the end of each cycle. Before commencing, a new cycle of treatment the participant must meet the parameters set in the eligibility criteria (including laboratory results), given in Table 1. Participants will continue on the trial regimen until disease progression, death, unacceptable toxicity or withdrawal of consent, whichever is sooner.

Participants randomised to CD alone may switch to receive ICD, according to the same schedule above, once disease progression is confirmed both by two TMG clinical members and two consecutive assessments (as defined by the IMWG response criteria), and eligibility has been confirmed as detailed in Table 2.

**Table 2:** MUK eight inclusion criteria for switching to ICD treatment

**Inclusion criteria for switching to ICD**	
●Randomised and treated in the CD only arm of the MUK eight trial ●Centrally confirmed disease progression by IMWG criteria. This must be confirmed by two consecutive assessments, local laboratory reports and confirmation of this must have been received via email from CTRU. ●ECOG performance status ≤ 2 ●Required laboratory values within 14 days prior to start of ICD treatment: ○ Platelet count ≥ 50 × 10^9^/L. Platelet count of 30–50 is acceptable if bone marrow aspirate shows tumour replacement of > 50%. Platelet transfusions to help patients meet these criteria are not allowed within 72 h prior to the blood sample to confirm eligibility to switch to ICD. ○ Absolute neutrophil count ≥ 1.0 × 10^9^/L. Growth factor support is **not** permitted within 14 days prior to randomisation ○ Haemoglobin ≥ 90 g/L. Blood support is permitted ○ ALT and / or AST ≤ 3 × upper limit of normal ○ Creatinine clearance ≥ 30 ml/min (using Cockcroft Gault formula) ○ Bilirubin ≤ 1.5 × upper limit of normal ●B2M performed within 14 days prior to the start of ICD treatment ●Female participants should avoid becoming pregnant and male participants should avoid impregnating a female partner. Both non-sterilised and sterilised females and males of reproductive age should use effective methods of contraception during the entire trial treatment (including treatment breaks) and up to 90 days after the last dose of trial treatment. Females of child bearing potential will require a negative pregnancy test to be performed.	

If a participant’s cyclophosphamide and/or dexamethasone doses have been reduced during the CD only phase of the trial, they will remain at the same dose when moving on to ICD.

### Trial assessments

Written informed consent must be obtained prior to the commencement of trial-specific assessments, and baseline assessments are to be performed within 14 days prior to randomisation. The required baseline assessments include a physical examination, ECOG performance status, medical history, ISS stage and ECG, as well as haematology, biochemistry and bone marrow sample tests. Participants must also complete the EQ-5D (5-level) and EORTC QLQ-C30 quality of life questionnaires prior to randomisation.

Assessments to be carried out within 48 h prior to the start of each cycle of treatment include a physical examination, peripheral neuropathy assessment, ECOG performance status, recording of adverse reactions and concomitant medications, and haematology and biochemistry tests. Participants will also complete the EQ-5D (5-level), EORTC QLQ-C30 and Resource Use questionnaires every 3 cycles. Laboratory results must be available and reviewed by the clinical team before dosing on day 1 of each cycle of treatment. Full blood counts will also be performed on days 8, 15 and 22 of cycles 1–3, and for the first 3 cycles of ICD following CD treatment. Blood samples for central investigations will be taken in cycle 4. Haematology and biochemistry results must remain within the parameters outlined in the eligibility criteria to proceed with each cycle of treatment.

End of treatment assessments, at the end of initial randomised treatment (CD or ICD) and at the end of the switch to ICD treatment, include a physical examination, peripheral neuropathy assessment, ECOG performance status, recording of adverse reactions and concomitant medications, and haematology, biochemistry and bone marrow tests. Participants will also complete the three quality of life questionnaires as before.

All participants will be followed up 4-weekly post the end of treatment until second disease progression. Follow-up will involve a response assessment and reporting of adverse reactions and SAEs will occur until 60 days post the last dose of trial treatment. Participants will be asked to complete the questionnaires at 3-month intervals until second disease progression. Participants who switch to ICD after treatment with CD will be followed up for 60 days post the end of treatment for toxicity (even if they stop treatment for (second) disease progression). After first disease progression, all participants will be followed up for a minimum of 12 months post-randomisation for survival only, until the time of final analysis.

### Data collection, management and monitoring

Participants will be identified by hospital sites during haematology clinic visits, and consent forms will be collected and checked to ensure all participants are consented fully. All data collected will be anonymised with a participant trial ID and sent with date of birth and initials to ensure accurate identification of participants for data entry. Data entry will be performed from paper Case Report Forms (CRFs) completed by each hospital site by internal staff and stored on the secure trial database accessed only by authorised members of CTRU staff. EORTC EQ-5D and QLQ-C30 will be used to collect quality of life data, in accordance with the EORTC instructions provided, therefore deemed valid and reliable.

Data management processes are fully documented in a trial work instructions document. Data will be 100% data checked for all items relating to trial endpoints and safety, i.e. doses of treatment and adverse reactions, for data quality. For any participant reaching the end of the trial on the CD arm and switching to the ICD arm, laboratory reports will be collected to verify this.

Adverse reactions will be collected on the CRF and 100% checked and data managed in the hope that this will ensure the data is accurate. Serious adverse events and suspected unexpected serious adverse events will be collected in real time with all being reviewed by the Chief Investigator within 24 h to guard against harm coming to the patient. Bleeding events will be closely monitored for the first 5 patients receiving ixazomib due to concerns regarding a low platelet count at inclusion.

### Statistical methods and analysis

For all statistical analyses, the analysis population (participants that are evaluable for the primary endpoints), as well as the safety population (participants that are evaluable for the toxicity and safety endpoints), are defined as those who receive at least one dose of any trial treatment. Toxicity and safety endpoints will be assessed according to the treatment actually received whilst effectiveness endpoints will be assessed by treatment the patient is randomised to. For example, patients on the ICD arm that do not receive ixazomib will be analysed together with the ICD patients that have received ixazomib.

#### Interim analysis

One formal interim efficacy analysis is planned for when 70% of the PFS events (139 events) have been observed, to assess for superiority of ICD in terms of PFS, and is estimated to take place at the same time as completing recruitment. This interim analysis is detailed in a Data Monitoring and Ethics Committee (DMEC) Interim Statistical Analysis Plan, and if, at the time of this interim analysis, ICD is found to be significantly superior when compared to CD, the DMEC may report to the Trial Steering Committee (TSC) with a recommendation of early release of data, rather than a recommendation of early trial closure. If the timing of this interim analysis falls before the end of recruitment, the DMEC may report to the TSC with a recommendation of early trial closure if appropriate.

#### Primary endpoint analysis

The primary endpoint analysis will compare the ICD arm to the CD arm in terms of PFS, with a null hypothesis of no difference between ICD and CD, and an alternative hypothesis of a difference between ICD and CD with ICD expected to be superior to CD. PFS curves will be calculated using the Kaplan-Meier method and the median PFS estimates and PFS estimates at 6 and 12 months with corresponding 95% confidence intervals will be presented by treatment group. A log-rank test, stratifying for the minimisation factors, will be used to compare PFS between the treatment groups.

The proportional hazards assumption will be assessed by plotting the hazards over time for each treatment arm, in addition to using the methods of Lin et al. [15] to check the adequacy of the Cox regression model. If the assumptions for Cox’s proportional hazards (Cox PH) model can be verified, a Cox PH model will be used. This model will adjust for the minimisation factors and other important prognostic factors identified by the TMG, in order to identify factors predictive of PFS between the treatment arms. Parameter estimates, standard errors, hazard ratios and 95% CIs, and *p* values will be presented for treatment and all other covariates included in the model.

#### Secondary endpoint analysis

For secondary endpoint analyses, maximum response is defined as the proportion of participants achieving each of the response categories sCR, CR, VGPR, PR, MR, SD or PD as their maximum response to initial treatment. Logistic regression adjusting for treatment group and minimisation factors will be used to predict the proportion of patients achieving at least a PR, and ordered logistic regression will be used to analyse the proportion of patients in each maximum response category, where appropriate (i.e. should the number of events in each maximum response level be sufficient).

The Kaplan-Meier method and log-rank test will be used to summarise the difference between treatment arms in terms of time to maximum response, duration of response and overall survival. If the Cox PH assumptions are confirmed as valid, a Cox PH model will also be fitted for these endpoints, in addition to the time to progression endpoint, provided no competing risk events have occurred. Residuals and predicted values produced from any multivariate models will be examined to assess the assumptions of the model. Competing risk analyses using cumulative incidence functions will be conducted for time to progression and time to maximum response endpoints. For these endpoints respectively, patients that have died with no previous evidence of disease progression and patients that have died prior to achieving a maximum response will be classed as having had a competing risk. Those not evaluable for maximum response (i.e. those with no response assessments following commencement of treatment) will be summarised as ‘no maximum response’ and excluded from analyses.

The cost-effectiveness analysis will consist of a decision model analysis informed by the trial data (patient reported resource use and the EQ-5D-5L) along with data from the wider literature and other sources where necessary. The primary analysis will be conducted from the healthcare provider perspective and report cost per quality-adjusted life year (QALY) gain over a lifetime horizon. Deterministic and stochastic sensitivity analyses will be performed. The analysis will be outlined in full in a health economics analysis plan ahead of analysis commencing.

Quality of life (QoL) will be summarised descriptively for each post-randomisation time-point using bar charts, box plots, plots of mean QoL over time and summary tables. Differences between treatment groups will also be obtained using a multi-level repeated measures model accounting for data at all post-baseline time points, assuming missing data at random (MAR). Missing data patterns will be examined, and sensitivity analyses using different missing data assumptions will be performed where appropriate, as described below.

A summary of the main comparisons and analysis methods for each secondary endpoint are given in Table 3.

**Table 3:** MUK eight secondary and exploratory endpoint analysis

Secondary (S) and exploratory (E) endpoints	Analysis method(s)
S(i) Proportion of patients achieving at least Partial Response	[D1] Logistic regression
S(ii) Proportion of patients in each maximum response category	[D1] Ordered logistic regression
S(iii) Time to progression	[D1] CIF and log-rank test, Cox PH
S(iv) Time to maximum response	[D1] Kaplan-Meier and log-rank test, Cox PH, CIF
S(v) Duration of response	[D1] Kaplan-Meier and log-rank test, Cox PH
S(vi) Overall survival	[D1] Kaplan-Meier and log-rank test, Cox PH
S(viii) Treatment compliance	[NFT] Descriptive summaries
S(ix) Safety and toxicity	[NFT] Descriptive summaries
S(x) Cost-effectiveness	[HE] Cost-utility analysis, cost effectiveness acceptability curves
S(xi) Quality of life	[D1] Multi-level repeated measures model
E(i) To identify possible biomarkers of response to ICD	[NFT] Descriptive summaries
E(ii) To estimate PFS2 for those that switch to ICD following CD treatment	[NFT] Kaplan-Meier and [D2] exploratory analyses: Kaplan-Meier and log-rank test, Cox PH
E(iii) To estimate PFS Switch for those that switch to ICD following CD treatment	[NFT] Kaplan-Meier and [D3] exploratory analyses of the ratio of PFS from switch against PFS on CD pre-switch
E(iv) Proportion of patients achieving at least Partial Response for ICD following CD treatment	[NFT] Descriptive summaries
E(v) Proportion of patients in each maximum response category of ICD following CD treatment	[NFT] Descriptive summaries
E(vi) Time to maximum response for ICD following CD treatment	[NFT] Kaplan-Meier
E(vii) Duration of response for ICD following CD treatment	[NFT] Kaplan-Meier
E(viii) Treatment compliance for ICD following CD treatment	[NFT] Descriptive summaries
E(ix) Safety and toxicity for ICD following CD treatment	[NFT] Descriptive summaries

*D1* differences between treatment arms (ICD vs CD), *D2* differences between treatment arms (ICD vs ICD Switch after progression on CD), *D3* differences between treatments (CD vs ICD Switch after progression on CD), *NFT* no formal statistical testing, *HE* health economic analysis, *Cox PH* Cox’s proportional hazards model, *CIF* cumulative incidence function curves for competing risk analysis

Sensitivity analyses for PFS and OS will be performed using imputed dates if there are > 5% of participants with a completely missing date of death/progression. Sensitivity analyses will also be conducted if there are > 5% of partially missing dates, using the earliest and latest possible day of the month. Sensitivity analyses for QoL may be carried out using methods such as multiple imputation, pattern-mixture multi-level models categorising participants into strata based on clinical information which is believed to represent the reasons for missing data (assuming MAR data conditional upon participants’ clinical data), and pattern mixture models for bivariate (baseline and 9 week) data fitted using a variety of restrictions reflecting the missing data pattern ranging from complete case missing variable restriction (MAR) to Brown’s protective restriction (assuming data are missing not at random (MNAR)).

PFS and OS will also be analysed adjusting for baseline Charlson Comorbidity Index [16] score (0, 1–2, > 2), which is calculated by assigning scores to each participant’s age and any comorbidities they exhibit, then summing the total of these scores. By including the Charlson Comorbidity Index scores in the Cox’s proportional hazards model for PFS and OS, this will determine the prognostic value of baseline Charlson index score on these outcomes. An interaction between baseline Charlson index score and treatment will also be included in the Cox proportional hazards models to assess the impact of the baseline score on treatment’s effect on the outcome. Deliverability of planned treatment according to baseline Charlson index score will be assessed by summarising the treatment compliance and toxicity endpoints according to participants’ baseline score (0, 1–2, > 2).

Exploratory analyses to investigate biomarkers of response will also be performed. These analyses will be detailed in full in a separate statistical analysis plan prior to any analysis undertaken.

Further exploratory analyses, detailed in Table 3, will be conducted for the switch phase of the trial, where patients on the CD arm may receive ICD treatment after confirmed progression on CD. These will include PFS2, PFS Switch, treatment compliance, and safety and toxicity. The definitions of PFS, PFS2 and PFS Switch are visualised in Fig. 2.

#### Frequency of analyses

A DMEC (made up of at least two clinicians and one statistician that are independent of the study team) will be set up to review data on safety, activity, protocol adherence and recruitment. The DMEC will review safety data for all participants entered into the trial after 12 participants have been recruited, and 3-monthly thereafter. Interim reports containing safety data, protocol adherence and recruitment will be presented to the DMEC in strict confidence at, at least, yearly intervals. This committee, in light of the interim data, and any advice and evidence they wish to request, will if necessary report to the TSC if there are any concerns regarding the activity or safety of the trial treatments.

The DMEC may also report to the TSC, with a recommendation of early release of data, should ICD be found to be significantly superior to CD during the interim analysis (once 70% of PFS events have been observed).

Final analysis will take place in two stages. Analysis of the primary endpoint will take place when at least 198 PFS events have been observed. At this time, all other endpoints relating to the first ‘phase’ of the study, with the exception of OS, will be analysed. A decision will be made based on the overall number of deaths observed as to whether OS will be analysed at the same time as PFS, or whether additional follow-up is required prior to analysis. Analyses relating to the switch phase of the study will take place after a minimum of 6 months follow-up after the last CD participant to switch treatments has started ICD treatment.

## Discussion

Despite advances in treatment options and improvements in survival rates over recent years, the overall prognosis for MM patients remains poor. Most patients will respond to initial treatment, potentially entering a plateau phase for a number of years, but relapse is inevitable. Subsequent response can be obtained by re-treating with the same or different regimens; however, some patients will become refractory to existing standard treatments. Ixazomib offers the opportunity to further investigate the value of proteasome inhibition in the treatment of RRMM whilst carrying the benefits of oral administration.

Two phase I studies have already investigated ixazomib in the RRMM setting [3, 5, 17]. These studies both incorporated a dose escalation phase followed by a dose expansion phase and indicated that ixazomib is well tolerated, with manageable toxicities. Dose limiting toxicities seen in these studies were skin rash, thrombocytopenia, nausea, diarrhoea, vomiting and erythema multiforme. Additionally a phase II study has investigated the safety and efficacy of ICD in patients with RRMM [18]. This study demonstrated a toxicity profile consistent with that seen in ixazomib in combination with lenalidomide and dexamethasone, whilst the combination appeared to have activity in RRMM patients. The promising clinical activity demonstrated in these studies, with durable responses and disease control, prove that further investigation of the anti-tumour effect of this drug in RRMM patients is warranted, especially given that no trials comparing CD with the combination of ixazomib and CD have been completed at present. As of today, there has been no evidence of impact on trial recruitment of ixazomib (Ninlarao) being available through the Cancer Drugs fund in England and SMC in Scotland. Ixazomib is not accessible in Northern Ireland. We therefore believe that demonstration of an improvement in PFS and overall survival for patients treated with ICD compared to CD would act as supporting evidence for the use of ixazomib in the UK.

## Data Availability

Not applicable—this is a protocol paper outlining the study being conducted.

## Abbreviations

ASCT: Autologous stem cell transplant
CD: Cyclophosphamide and dexamethasone
CR: Complete response
CTN: Clinical Trials Network
CTRU: University of Leeds Clinical Trials Research Unit
DMEC: Data Monitoring and Ethics Committee
ECG: Electrocardiogram
ECOG: Eastern Cooperative Oncology Group
EORTC: European Organisation for Research and Treatment of Cancer
EQ-5D: EuroQol five-dimension scale
QLQ-C30: Quality of life questionnaire
ICD: Ixazomib, cyclophosphamide and dexamethasone
IMWG: International Myeloma Working Group
ISS: International Staging System
MM: Multiple myeloma
MR: Minimal response
MUK: Myeloma UK
OS: Overall survival
PFS: Progression-free survival
PFS2: Second progression-free survival measured from randomisation
PFS Switch: Progression-free survival measured from the point of switching to second progression
PR: Partial response
RRMM: Relapsed/refractory multiple myeloma
sCR: Stringent complete response
SD: Stable disease
TMG: Trial Management Group
TSC: Trial Steering Committee
VGPR: Very Good Partial Response
